# Isolation and characterization of lactic acid bacteria with potential probiotic activity and further investigation of their activity by α-amylase and α-glucosidase inhibitions of fermented batters

**DOI:** 10.3389/fmicb.2022.1042263

**Published:** 2023-01-23

**Authors:** Sujay S. Huligere, V. B. Chandana Kumari, Taha Alqadi, Saurabh Kumar, Charley A. Cull, Raghavendra G. Amachawadi, Ramith Ramu

**Affiliations:** ^1^Department of Biotechnology and Bioinformatics, School of Life Sciences, JSS Academy of Higher Education and Research, Mysore, Karnataka, India; ^2^Department of Biology, Adham University College, Umm Al-Qura University, Makkah, Saudi Arabia; ^3^Kerry Food Center, Inc., Beloit, WI, United States; ^4^Midwest Veterinary Services, Inc., Oakland, NE, United States; ^5^Department of Clinical Sciences, College of Veterinary Medicine, Kansas State University, Manhattan, KS, United States

**Keywords:** lactic acid bacteria, gastrointestinal tract, anti-hyperglycaemic, probiotics, α-Glucosidase, α-Amylase

## Abstract

Probiotic microbiota plays a vital role in gastrointestinal health and possesses other beneficial attributes such as antimicrobial and antibiotic agents along with a significant role in the management of diabetes. The present study identifies the probiotic potential of *Lactobacillus* spp. isolated from three traditionally fermented foods namely, jalebi, medhu vada, and kallappam batters at biochemical, physiological, and molecular levels. By 16S rRNA gene amplification and sequencing, the isolates were identified. A similarity of >98% to *Lacticaseibacillus rhamnosus* RAMULAB13, *Lactiplantibacillus plantarum* RAMULAB14, *Lactiplantibacillus pentosus* RAMULAB15, *Lacticaseibacillus paracasei* RAMULAB16, *Lacticaseibacillus casei* RAMULAB17, *Lacticaseibacillus casei* RAMULAB20, and *Lacticaseibacillus paracasei* RAMULAB21 was suggested when searched for homology using NCBI database. Utilizing the cell-free supernatant (CS), intact cells (IC), and cell-free extract (CE) of the isolates, inhibitory potential activity against the carbohydrate hydrolyzing enzymes α-glucosidase and α-amylase was assessed. CS, CE, and IC of the isolates had a varying capability of inhibition against α-glucosidase (15.08 to 59.55%) and α-amylase (18.79 to 63.42%) enzymes. To assess the probiotic potential of seven isolates, various preliminary characteristics were examined. All the isolates exhibited substantial tolerance toward gastrointestinal conditions and also demonstrated the highest survival rate (> 99%), hydrophobicity (> 65%), aggregation (> 76%), adherence to HT-29 cells (> 84%), and chicken crop epithelial cells suggesting that the isolates had a high probiotic attribute. Additionally, the strains showed remarkable results in safety assessment assays (DNase and hemolytic), and antibacterial and antibiotic evaluations. The study concludes that the lactic acid bacteria (LAB) characterized possesses outstanding probiotic properties and has antidiabetic effects. In order to obtain various health advantages, LAB can be utilized as probiotic supplements.

## Introduction

Diabetes mellitus (DM) claims more lives than several other disorders known to mankind. It is found that one person dies from DM every 10 s, making it a significant contributor to death from a long-term illness. Due to the staggering rise in diabetes worldwide, it has now been established as a global epidemic causing a huge burden to the healthcare and economy of most developed and developing countries. Recent predictions worldwide indicate a rise in DM in adults reaching nearly 380 million by 2025 from 194 million in 2010, with the most affected countries being India, China, and the United States (Kaul et al., 2012). Diabetic individuals lose control over their blood glucose levels, which leads to both short and long-term complications owing to the presence of glucose in circulation for an extended period of time. DM is a common disorder with little knowledge of its etiology, which is most likely due to a number of genetic and environmental factors contributing to the heterogeneity of type 2 diabetes (Sreepathi et al., 2022). The most prospective targets for the treatment of DM include inhibition of the enzymes α-glucosidase and α-amylase which constitute a means to prevent hyperglycemia. The two enzymes are involved in the breakdown of complex carbohydrates into their constituents in the proximal intestinal brush border. If they are inhibited, it leads to a delay in the absorption of carbohydrates and reduced postprandial glucose excursions. As a result of the increase in blood sugar, there is a reduction in postprandial insulin secretion (Fonseca and John-Kalarickal, 2010). There are several pharmacological drugs available that can be used to intensify the treatment. These include insulin sensitizers-biguanide, metformin and thiazolidinediones, insulin secretagogues-sulfonylureas, and non-sulfonylurea secretagogues, (GLP-1) agonists, DPP4 inhibitors, and pramlintide, an analogue of the peptide amylin that the beta cell co-secretes with insulin, is commonly recommended for use with insulin in both type 1 and type 2 diabetes (Alam et al., 2014). The consumption of these pharmacological drugs as therapy has its own long-term side effects such as renal impairment, cardiovascular diseases, loss of appetite, fluid retention, frequent gastrointestinal (GI) tract infection, etc. In relation to this, the gut microbiota is essential for maintaining a number of diabetic metabolisms (Leu and Zonszein, 2010). Altering the gut flora to attain or maintain a favorable condition is advisable for the improvement of the host’s health. When compared to the other commercially accessible medications, using this as therapy has lesser-known negative effects. These live microorganisms that are introduced into the body for their beneficial properties are commonly referred to as probiotics (Kumari et al., 2022a). A change in the GI ecology can be facilitated by the administration of these live microorganisms. There is substantial evidence that probiotics can interact with gut-associated lymphoid tissue, restrict the growth and adhesion of potentially pathogenic organisms, and influence both mucosal immunity and systemic immunity (Qin et al., 2005). The incorporation of such probiotic microbiome through fermented food sources has been demonstrated to be economically efficient worldwide because the raw ingredients used are easily accessible, have low economic value, and appetizing in their raw state (Soni and Dey, 2014). The process of fermentation has been practiced worldwide for enhancing nutrients and making them more accessible while retaining and boosting the amounts of many beneficial bioactive compounds (Guan et al., 2021). It also enhances the product’s sensory qualities in addition to eliminating unwanted components. The macro-and micronutrient balance of fermented foods made from cereals could be improved by co-fermenting cereals and legumes to create inexpensive, protein-rich diets (Gupta and Tiwari, 2014). It is interesting to note that indigenous communities and ethnic groups have been practicing lactic acid fermentation to preserve seasonal and perishable vegetables and fruits (Nag and Das, 2013). In developing nations, backslopping and spontaneous fermentation are still used to produce fermented foods and beverages today, while large-scale manufacturing of fermented foods has grown to constitute a significant sector of the global food business in wealthier nations (Wang et al., 2018). The great gourmet attributes of traditionally fermented foods are valued by consumers worldwide for their exceptional health-benefiting qualities (Kariyawasam et al., 2021).

Contrarily, the fermentation of traditionally fermented foods is commonly brought on by naturally occurring, wild varieties of LAB that are derived from the raw material, the processing equipment, or the environment and that start the fermentation process in the absence of a commercial starter (Franz et al., 2014). It is also noted that pure strains isolated from the intricate ecosystems of traditionally fermented foods exhibit a wide range of metabolic activities that greatly differ from those of equivalent strains employed as bulk starters for commercial processes (Silva et al., 2004). Such findings emphasize the significance of the Designation of Protected Origin (DPO) for many of these goods, which is essential from an economic perspective as they let small-scale fermentation units survive in a world of ongoing globalization (da Silva Duarte et al., 2022).

In recent years, it has become popular to separate wild-type strains from conventional products to utilize them as starter cultures for food fermentation as they possess numerous benefiting attributes in enhancing the food grade levels (Carminati et al., 2010). Indian cuisine on a daily basis consists of a wide variety of traditionally fermented foods made of cereals like rice, wheat, ragi, etc., along with other various legumes that are consumed in large quantities. Idli, and dosa, are the main rice-based overnight fermented south Indian cuisines among the various options (Rao et al., 2013). Where such traditionally fermented food products are majorly fermented by a wild variety of LAB species (Satish Kumar et al., 2013). Lactic acid bacteria (LAB) are well-known probiotics worldwide; when consumed, LAB provides numerous health benefits for the host. Incorporation of these probiotics into the gut through consumption is one of the safest and most modern approaches aiding to their health benefits (Lye et al., 2017). With a long history of use and safe consumption in the development of fermented foods and beverages, the LAB species plays a key role in fermentation. Probiotics are habitually found in dairy products that have been fermented with lactic acid bacteria (LAB), particularly yogurt (Savaiano and Hutkins, 2021). During the production of yogurt, lactose is transformed into lactic acid by a yogurt culture until a final pH of 4.2 to 4.5 is reached. The pH may go to 4.0 after storage. *Lb. delbrueckii subsp. bulgaricus* is implicated in this unfavorable post-acidification, which results in an acidic and bitter flavour. Since these cells can only grow in the presence of actively lactose-fermenting *S. thermophilus* cells due to their protocooperation, lactose-negative mutations of *Lb. delbrueckii subsp. bulgaricus* allow for the creation of mild yogurt (Gilbert et al., 1996). Due to the actions of endogenous milk enzymes as well as the proteolytic and lipolytic activities of LAB present in the cheese, many aromatic compounds are produced during cheese maturation (Fox and McSweeney, 1996). LAB enhances the flavor and aroma of fermented commodities. They create aromatic molecules from amino acids after further bioconversion, acidify the meal, giving it a tart lactic acid flavor, and frequently engage in proteolytic and lipolytic activities (Yvon and Rijnen, 2001).

It has been a technological challenge for the food industry to develop probiotic dairy and fermented products to maintain the stability of LAB and probiotics from manufacturing to consumption. The market preference for foods that are fresh, safe, delicious, low in sugar, fat, and salt, and simple to prepare seems to be in conflict with the demand for less processing and additive use. The connection between food and health is a major concern for consumers today. As a result, the market for foods with health-promoting qualities, often termed functional foods, has experienced an impressive expansion in recent years (Granato et al., 2020). These market shifts put pressure on the food industry to find alternatives. In food fermentation, one of the most important areas for intervention appears to be at the level of the starter culture. The commercial availability of novel intriguing starter cultures is constrained, and industrial starter cultures regrettably lack the required traits for product diversity (Tsuda, 2018). An improved understanding of the food microorganisms’ genomes and metabolomics gives up possibilities through molecular techniques. The probiotic LAB isolated from such traditional fermented sources has proven to exhibit anti-diabetic, anti-microbial, and antibiotic activities (Kim and Lim, 2019). In addition to the health-promoting effects, LAB isolates must exhibit certain characteristics to prove their probiotic attributes. Initial traits of the probiotic bacteria include bile and acid tolerance, adhesion to host epithelial cells, and safety assessment. However, the benefits of this probiotic biotherapy have been poorly elucidated (Harun-Ur-Rashid et al., 2007). In this regard, the present study was designed to determine the antidiabetic attributes of the probiotic LAB species isolated from traditionally fermented - jalebi, medhu vada, and kallappam batters (Muganga et al., 2015; Alkalbani et al., 2019; Cai et al., 2019; Jeong et al., 2021). The southern part of India has seen various health benefits from consuming these foods regularly, but the findings are poorly elucidated. Therefore, our study mainly focuses on the evaluation of the probiotic attributes and the ability to inhibit the carbohydrate hydrolyzing enzymes α-glucosidase and α-amylase of such traditionally fermented sources (Kwun et al., 2020). The ideally discovered cultures can also be used as starting cultures in the food fermentation sector which later can improve food safety and/or provide a variety of organoleptic, technical, nutritional, or health benefits. Implementing these chosen strains as starter cultures or co-cultures in fermentation processes can assist in achieving *in situ* expression of the desired attribute, preserving a completely natural and healthy result.

## Materials and methods

### Materials

*Lactobacillus* de Man, Rogosa, and Sharpe (MRS) agar and broth, oxgall salt glycerol, phenol, NaCl, xylene, deoxyribonuclease (DNase) agar medium, blood agar medium with 5% (w/v) sheep blood, ABTS, DPPH, and, antibiotic susceptibility disc were procured from HiMedia Laboratories Pvt. Ltd., Mumbai, India. The pathogens namely, *Bacillus subtilis* MTCC 10403, *Escherichia coli* MTCC 4430, *Pseudomonas aeruginosa* MTCC 424, *Micrococcus luteus* MTCC 1809, and *Salmonella typhimurium* MTCC 98 were procured from the Microbial Type Culture Collection and Gene Bank (MTCC), Chandigarh, India.

### Bacterial isolation from the fermented batters

Rice and pulse-based batters of various traditional fermented foods like jalebi, medhu vada, and kallappam were freshly prepared, in the month of April at room temperature (37°C) in Mysuru, Karnataka, India. For the jalebi batter, a 1:4 proportion of chickpea flour and all-purpose flour was mixed together and a pinch of baking soda was added to enhance the fermentation process. For medhu vada 1:6 proportion of rice flour and soaked black gram was blended in a mixer to batter consistency, whereas for Kallappam a 1:8:4 proportion of toddy, semolina, and coconut paste, respectively, was mixed into a thick batter consistency. All the above batters were allowed to ferment overnight. No additional cultures were added into the batters for fermentation enhancement. The overnight fermented batters were then serially diluted (phosphate saline 0.1 M, pH 7.2) and pour-plated on a solidified MRS agar plate. Anaerobic incubation was performed at 37°C for 24–48 h. The colonies with various morphological characteristics were selected and pure cultures were streaked onto the MRS agar plate. The colonies were isolated, sub-cultured in MRS broth, and stored at 4°C. For further investigation, the isolated strains were prepared as an extract (CE), and supernatant (CS) as per the methodology mentioned by Jo et al. (2021).

### Biochemical characterization

The isolates were preliminarily characterized in accordance with the guidelines from Bergey’s Manual of Determinative Bacteriology. All initially screened isolates were tested for tolerance at different temperatures (4, 10, 37, 45, and 50°C), salt (2, 4, 8, and 10%), pH (2, 4, 6, and 8) concentrations, and carbohydrate fermentation against 10 sugars (Table 1) and evaluated (Fitriani et al., 2021).

**Table 1:** Characteristics of LAB strains isolated from fermented batters in terms of phenotypic, biochemical, and fermentation capacity.

Isolates							
Tests	RAMULAB 13	RAMULAB 14	RAMULAB 15	RAMULAB 16	RAMULAB 17	RAMULAB 20	RAMULAB 21
Gram staining	Positive						
Catalase	Negative						
Morphology	Short Rod	Rod	Short Rod	Rods	Short Rod	Short Rod	Rod
***Biochemical parameters***							
Methyl Red	**+**	**+**	**+**	**+**	**+**	**+**	**+**
Voges Proskauer	**−**	**−**	**−**	**−**	**−**	**−**	**−**
Indole	**−**	**−**	**−**	**−**	**−**	**−**	**−**
Citrate	**−**	**−**	**−**	**−**	**−**	**−**	**−**
Starch Hydrolysis	**−**	**−**	**−**	**−**	**−**	**−**	**−**
Gelatin Liquification	**−**	**−**	**−**	**−**	**−**	**−**	**−**
***Probiotic properties***							
*Temperature-related growth (°C)*							
4	**−**	**−**	**−**	**−**	**−**	**−**	**−**
10	**−**	**−**	**−**	**−**	**−**	**−**	**−**
37	**+**	**+**	**+**	**+**	**+**	**+**	**+**
45	**−**	**−**	**+**	**−**	**−**	**+**	**+**
50	**−**	**−**	**−**	**−**	**−**	**−**	**−**
*Salt–related growth (%)*							
2	**+**	**+**	**+**	**+**	**+**	**+**	**+**
4	**+**	**+**	**+**	**+**	**+**	**+**	**+**
8	**−**	**−**	**−**	**−**	**−**	**−**	**−**
10	**−**	**−**	**−**	**−**	**−**	**−**	**−**
*pH-related growth*							
2	**+**	**+**	**+**	**+**	**+**	**+**	**+**
4	**+**	**+**	**+**	**+**	**+**	**+**	**+**
6	**+**	**+**	**+**	**+**	**+**	**+**	**+**
7.4	**+**	**+**	**+**	**+**	**+**	**+**	**+**
*Carbohydrates fermentation*							
Lactose	**+**	**+**	**+**	**+**	**+**	**+**	**+**
Glucose	**+**	**+**	**+**	**+**	**+**	**+**	**+**
Maltose	**+**	**+**	**+**	**+**	**+**	**+**	**+**
Sucrose	**+**	**+**	**+**	**+**	**+**	**+**	**+**
Mannitol	**+**	**+**	**+**	**+**	**+**	**+**	**+**
D-xylose	**−**	**−**	**+**	**+**	**−**	**−**	**−**
L-xylose	**−**	**−**	**−**	**−**	**−**	**−**	**−**
Galactose	**+**	**+**	**+**	**+**	**+**	**+**	**+**
Arabinose	**−**	**−**	**−**	**−**	**−**	**−**	**−**
Starch	**−**	**−**	**−**	**−**	**−**	**−**	**−**

“+” indicates presence and “−” indicates absence.

### Evaluation of probiotic attributes *in vitro*

#### Acid and bile salt

The methodology described by Pan et al. (2009) was used to execute the acid and bile salt tolerance studies, with a few minor adjustments. One hundred microliter of LAB isolates were inoculated into the MRS broth (with pH 2 and (0.3 and 1%) oxgall salt) and incubated at 37°C. The samples were enumerated at 0,2, and 4 h of incubation. The following formula was used to determine the survival rate (%):


$$\begin{array}{l} \text{Survival rate}\;\left(\%\right)\\ =\left[\text{Biomass at time}\left(t\right)/\text{Biomass at initial time}\left(0\right)\right]\times 100. \end{array}$$

#### Simulated gastrointestinal conditions

When ingested, the isolates must withstand the gastric and intestinal conditions for up to 3 and 8 h, respectively in accordance with a healthy digestive process. The assay was performed by the methodology mentioned by Musikasang et al. (2009) with slight modification. Pepsin (3 g/l of PBS, pH 3; 1:3000 AU/mg, Sisco Research Laboratories Pvt. Ltd., Mumbai, India) and trypsin (1 g/l of PBS, pH 8; 2000 U/g, Sisco Research Laboratories Pvt. Ltd., India) were dissolved to prepare simulated gastric and simulated intestinal juice conditions. Under gastrointestinal conditions, the isolates (10^9^ CFU/ml) were subsequently inoculated into the juices in an *in vitro* condition. The tolerance of the isolates towards gastrointestinal conditions was evaluated using viable colony counts. The following equation was used to determine the percentage of survival (%):


$$\text{Survival rate}\;\left(\%\right)=\left[\log\;\text{CFU}\;N_{1}/\log\;\text{CFU}\;N_{0}\right]\times 100.$$

where *N*_1_ = Number of viable cells after treatment and *N*_0_ = Number of viable cells before treatment.

#### Phenol

The experimental approach by Jena et al. (2013) was used to evaluate the viability and survival rate of the isolates in the presence of phenol solution by inoculating the LAB isolated (10^8^ CFU/ml) in MRS broth containing 0.4% phenol (24 h, 37°C). By serial dilution onto the MRS agar plate, the bacterial enumeration was performed at 0 h and 24 h of the experiment.

### Cell adherence assays

#### Cell surface hydrophobicity

The LAB isolates were examined against the polar solvent xylene at 600 nm to understand their cell surface interaction using the previously available approach by Li et al. (2020). In order to aid the separation of the two phases, 1 ml of xylene and 3 ml of LAB cell suspension (10^8^ CFU/ml) were mixed in a test tube and allowed to settle for 2 h at 37°C. The absorbance of the aqueous phase was calculated by the equation given below:


$$\text{Hydrophobicity}\;\left(\%\right)=\left[1–\left(A/A_{0}\right)\right]\times 100.$$

where, *A* = final absorbance of the aqueous phase, *A*_0_ = initial absorbance.

#### Autoaggregation

The autoaggregation of the isolates was performed as per the methods followed by Tareb et al. (2013) at an absorbance of 600 nm. Briefly, the 18 h cultured cells were harvested and resuspended in PBS (10^8^ CFU/ml) and evaluated for their ability to aggregate to the cells. Autoaggregation percentage was calculated for time intervals of 0, 2, 4, 6, 10, and 24 h using the equation:


$$\text{Autoaggregation}\;\left(\%\right)=\left[1–\left(A_{t}/A_{0}\right)\right]\times 100.$$

where, *A_t_* and *A*_0_ denote absorbance at the time “t” and “0” (initial), respectively.

#### Coaggregation

Cell suspension for LAB isolates was taken in the ratio of 2:1 was taken, each of the 5 pathogenic strains [(*Escherichia coli* MTCC 4430, *Pseudomonas aeruginosa* MTCC 98, *Bacillus subtilis* MTCC 10403), and *Salmonella typhimurium* (MTCC 98)] were mixed and incubated at 37°C for 2 h. At 600 nm, the combination’s absorbance was recorded and analyzed. The methodology was carried out as the early approach mentioned by Tatsaporn and Kornkanok (2020). The coaggregation percentage was expressed as:


$$\text{Coaggregation}\left(\%\right)=[\left(A_{L}+A_{P}\right)–A_{\text{mix}}/\left(A_{L}+A_{P}\right)\times 100.$$

where *A*_mix_ signifies [the absorbance of the LAB mixture + pathogen at 4 h], and *A_L_* + *A_P_* denotes [the absorbance of the LAB mixture + pathogen at 0 h].

#### Adherence to chicken crop epithelial cells

LAB adhesion to crop epithelial cells of chicken was investigated as given by Somashekaraiah et al. (2019) under *in vitro* conditions. LAB isolates and chicken crop epithelial cells (1 × 10^6^ cells/mL) were mixed at a 1:10 ratio and incubated for 1 h at optimum temperature. After incubation, the cells were centrifuged (3,000 rpm, 5 min) for the elimination of non-adherent bacterial cells. The pellets obtained were rinsed and resuspended in 100 μl PBS, stained using crystal violet, and viewed under the bright field microscope at 100X magnification.

#### HT-29 cell culture and development circumstances

According to the methodology performed by Jeong et al. (2021), the adhesion capacity of the seven isolates to HT29 cells (human colon cancer cell lines) was evaluated. The cells (passage #123–130, National Centre for Cell Science in Pune, Maharashtra, India) were grown in DMEM (25 mM, High media) with GlutaMAX (Gibco, United Kingdom) at 37°C in 5% CO_2_. A total of 100 g/ml of penicillin and streptomycin and 10% (v/v) FBS (Gibco, UK) were added to the medium as supplements. In a six-well culture plate, HT-29 cells were subcultured at1x10^5^ cells/mL and grown at 37°C in a humidified CO_2_ atmosphere until they reached 70% confluence in the cell medium. The culture medium was changed every alternate day. As a part of the adhesion test, isolates were grown (16 h, 37°C) in MRS broth. The cells were resuspended in DMEM medium at a concentration of 10^8^ CFU/ml and washed twice with PBS. To each well, 1 ml of bacterial suspension [incubated for 30 and 60 min at 37°C (5% CO_2_ atmosphere)] was added. The cells were lysed by adding 1 ml of 0.1% Triton-X solution (in PBS) and the non-adherent cells were removed by adding PBS. The solution containing the discharged bacterial cells was serially diluted and plated on MRS agar after 10 min at 37°C and incubated for 24 h. The percentage ratio of the initial number of bacteria implanted to that seeded following washing (CFU/mL) was used to determine its adhesion ability. The experiments were carried out in triplicates. The following equation was used to calculate the adhesion rate of the LAB strains:


$$\text{Adhesion rate}\left(\%\right)=\left(C/C_{0}\right)\times 100.$$

where, *C* = Number of adherent cells, *C*_0_ = Initial number of cells inoculated.

### Safety assessment

#### Antibiotic sensitivity

As per Clinical and Laboratory Standards Institutes (CLSI) guidelines 2018, the antibiotic susceptibility against LAB isolates (10^8^ CFU/ml) was evaluated by disc diffusion method for the following antibiotic discs–namely, clindamycin (2 mcg/disc), chloramphenicol (30mcg/disc), ampicillin (10 mcg/disc), gentamicin (10 mcg/disc), tetracycline (30 mcg/disc), kanamycin (30mcg/disc), rifampicin (5mcg/disc), vancomycin (30 mcg/disc), methicillin (5mcg/disc), erythromycin (15 mcg/disc), streptomycin (100 mcg/disc), cefixime (5mcg/disc), and azithromycin (15mcg/disc). The results were elucidated as resistant(R), susceptible (S), or moderately susceptible (MS) by comparison with the diameter of the zone of inhibition.

#### Hemolytic assay

Hemolytic activity of the isolated LAB strains was examined on blood agar plates (HiMedia, Mumbai, India; Halder et al., 2017). Based on the red blood cell lysis in the medium surrounding the colonies, the hemolytic activity of the isolated strains was assessed. The plates were observed for the appearance of a clear zone surrounding the colony for hemolytic reaction.

#### DNase activity

To evaluate the isolate’s capacity to produce deoxyribonuclease (DNase) enzymes, LAB isolates were streaked onto DNase agar plates (HiMedia, Mumbai, India). DNase activity was evaluated by the formation of the clear zone after incubation for 48 h at 37°C. A distinct zone surrounding the colonies found evidence of positive DNase activity (Boricha et al., 2019).

#### Antimicrobial activity

The antimicrobial activity of the isolates against pathogenic strains was assessed using the agar well diffusion method as per Arqués et al. (2015) with slight modifications. *Bacillus cereus* MTCC 10403, *Staphylococcus aureus* MTCC 1144*, Salmonella typhimurium* MTCC 98*, Escherichia coli* MTCC 443*, Pseudomonas aeruginosa* MTCC 424*, Klebsiella pneumonia, Micrococcus luteus* MTCC 1809*, Bacillus subtilis* MTCC 10403*, Pseudomonas florescens* MTCC 667, *and Klebsiella aerogenes (Enterobacter aerogenes)* MTCC 2822 were the test organisms. The pathogen (100 μl) was added to Luria Bertani agar (LB agar) plates. Wells were made on the solidified agar medium using a well borer for the treatment of LAB isolates. A 100 μl of 18 h overnight grown LAB isolates were poured into the well, allowed to dry, and incubated at 37°C for 24–48 h.

### Molecular identification

Molecular characterization provides a basis for the cultural and biochemical characterization of LAB isolates. The study was performed as per the method described by Agaliya and Jeevaratnam (2013) using the regions of 16S rRNA. Seven LAB isolates obtained in the present study were amplified and sequenced using specific primers targeting the 16S rRNA region. The obtained sequence was evaluated by BLAST analysis and a phylogenetic tree was constructed using MEGA X (Version 10.2.4, CA, United States) 0.1000 bootstrap consensus trees were used to create the phylogenetic tree with the highest likelihood. The Tamura-Nei model fits the best (Tamura et al., 2007). In order to automatically create the initial tree(s) for the heuristic search, the Neighbor-Joining and BioNJ algorithms were used on a matrix of pairwise distances.

### Antioxidant activities

#### Radical scavenging rate by DPPH assay

The methodology performed by Elfahri et al. (2014) was followed to conduct the assay. The radical scavenging activity of the cells at 10^3^, 10^6^, and 10^9^ CFU/ml was assessed using the DPPH (1,1-diphenyl-2-picrylhydrazyl) assay. To express the radical scavenging activity, the following equation was used.


$$\text{Scavenging rate}\;\left(\%\right)=1–\left[\left(A_{s}/A_{b}\right)\times 100\right].$$

where *A_S_* = absorbance of the reactants with the sample and *A_b_* = absorbance of the reactants without the sample.

#### Radical scavenging rate by ABTS assay

The method described by Soleymanzadeh et al. (2016) was used to estimate the radical scavenging rate of the cells at 10^3^, 10^6^, and 10^9^ CFU/ml by 2,2′-azino-bis (3-ethyl benzo-thiazoline-6-sulphonic acid) (ABTS) assay. The below-mentioned equation was used to compute the radical scavenging activity:


$$\text{Scavenging rate}\;\left(\%\right)=1–\left[\left(A_{s}/A_{b}\right)\times 100\right].$$

where *A_S_* = absorbance of the reactants with the sample and *A_b_* = absorbance of the reactants without the sample.

### Inhibitory activity of carbohydrate hydrolyzing enzymes

#### α-Glucosidase

The α-glucosidase inhibitory activity was performed with slight modifications as described by Reuben et al. (2019). α-glucosidase (ex. yeast, 100 U/mg) was used for inhibitory activity. The test samples (CS, CE, and IC – 100 μl) and 50 mM PBS buffer (pH 6.8) were mixed and incubated for 10 min. α-glucosidase (100 μl, 0.25 U/ml) was added and pre-incubated for 15 min at 37°C. Five millimeter pNPG (p-nitrophenol-D-glucopyranoside-100 μl) was added and re-incubated for 30 min at 37°C. The enzymatic reaction was stopped by adding 1,000 μl of 0.1 M Na_2_CO_3_ and the absorbance of 4-nitrophenol was measured at 405 nm. The percent inhibition was calculated as per the following formula,


$$\text{Inhibition}\;\left(\%\right)=1–\left[\left(A_{s}/A_{c}\right)\times 100\right].$$

where A_C_ denotes the absorbance of the reactants in the absence of the sample and A_S_ denotes the absorbance of the reactants when combined with the sample.

#### α-Amylase

The inhibitory potential of CS, CE, and IC against the α-amylase enzyme was evaluated according to the procedure of Ankolekar et al. (2012) with a minor modification. Briefly, porcine pancreatic α-amylase was used in the inhibition assay. CS, CE, and IC (500 μl) obtained from the isolates were mixed with 500 μl of 0.1 M PBS (containing 0.5 mg/ml of α-amylase solution, pH 6.4). The mixtures were incubated at 25°C for 10 min. At specified intervals after the pre-incubation, 1% starch solution (500 μl) prepared in 0.1 M PBS (pH 7.4) was added to each tube. The reaction mixtures were subsequently incubated at 25°C for an additional 10 min. The reaction was terminated using 1.0 ml of DNS reagent followed by incubation in a boiling water bath for 5 min. The tubes were cooled in order to bring it to room temperature. After adding 10 ml of distilled water to the reaction mixture, the absorbance was measured at 540 nm and the percent inhibition was calculated using the equation:


$$\text{Inhibition}\left(\%\right)=1–\left[\left(As/Ac\right)\times 100\right].$$

where, *A_C_* denotes the absorbance of the reactants in the absence of the sample, and *A_S_* denotes the absorbance of the reactants when combined with the sample.

### Statistical analysis

The experiments were carried out in triplicate. The standard deviation is displayed in error bars on graphs. Data examination was done using a one-way analysis of variance (ANOVA). *p* ≤ 0.05 was used to determine the significance of the differences.

## Results

### Preliminary characterization of LAB

A total of 24 isolates were identified from the three batters evaluated in the present study. Seven isolates were classified as LAB in accordance with their phenotypic traits. All of the isolates had a rod-like morphology, were Gram-positive, and did not produce catalase. Biochemical characteristics determined that the isolates were hetero-fermentative, with no gas liberation from glucose fermentation. At an optimum temperature of 37°C, all the isolates showed conventional growth, strains RAMULAB15, RAMULAB20, and RAMULAB21 were able to withstand a temperature of up to 45°C. All the isolates demonstrated optimum growth at salt concentrations of 2 and 4% in the media. At pH 2, 4, and 6, the isolates exhibited mild growth whereas showed optimum growth at pH 7.4. Lactose, glucose, maltose, sucrose, and mannitol could be fermented by all seven isolates (Table 1).

### Evaluation of probiotic attributes

#### Acid bile salt tolerance

**Figure 1:**
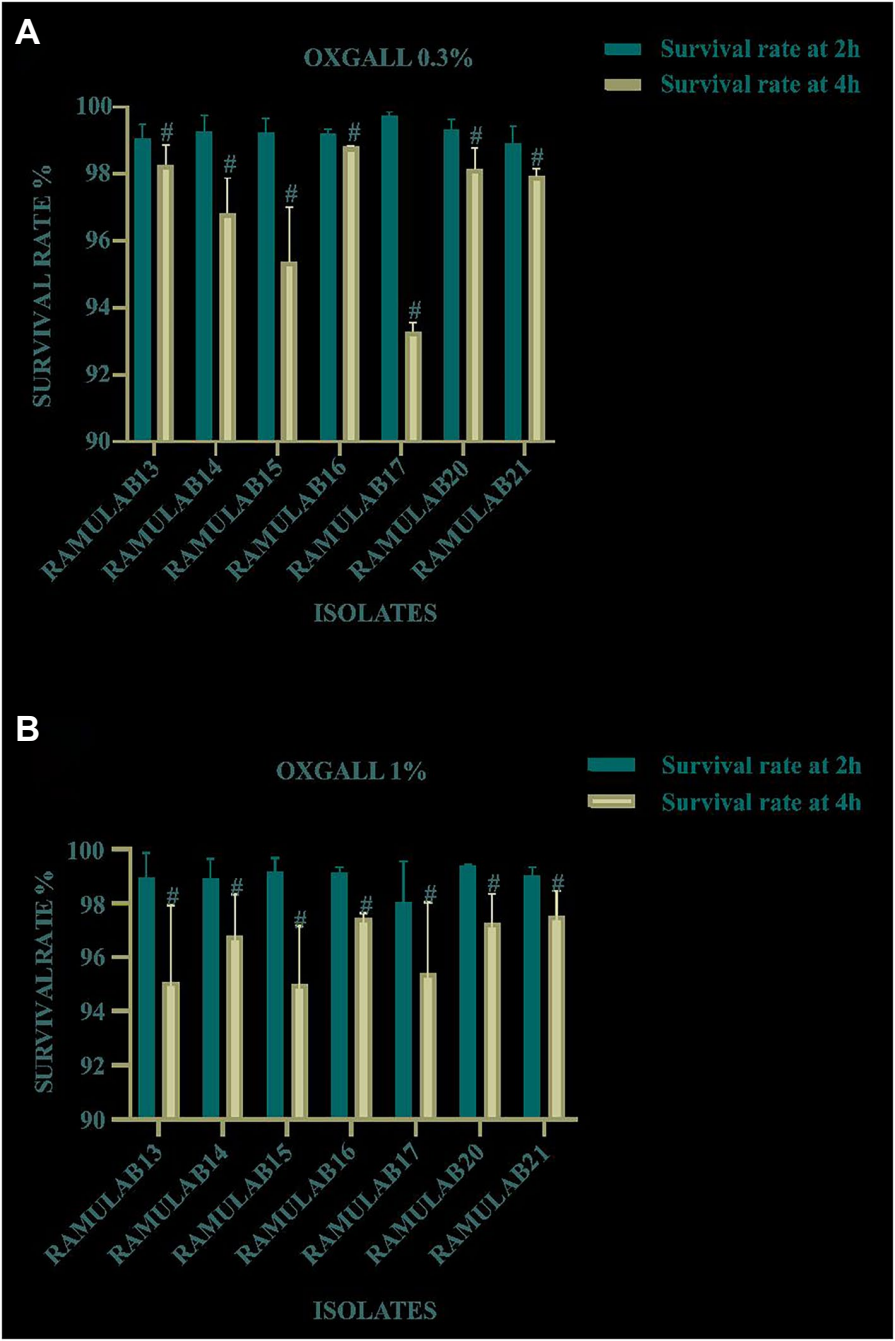
Survival of the isolates on MRS agar plates for 2 and 4 h at 37°C under circumstances of acidic pH 2 and **(A)** 0.3% and **(B)** 1% bile salt concentration. Data are presented as mean ± standard deviation. The Duncan multiple range tests revealed that the means of the survival rate with a 2 h time interval and superscripts (#) are substantially different from one another (*p* ≤ 0.05).

The survival rate of all the LAB isolates tested at pH 2 was assessed under bile conditions (0.3 and 1%) for the evaluation of acid and bile tolerance. Figures 1A,B displays the isolate’s capacity to survive at pH 2 and their tolerance to 0.3 and 1% of acid bile, respectively. After 4 h of incubation, for 0.3 and 1% of acid bile, the LAB strains exhibited the highest survival rate of 98 and 93%, respectively. Notably, all seven isolates demonstrated a high survival rate at a bile concentration of 0.3%.

#### Simulated gastrointestinal juice tolerance assay

**Figure 2:**
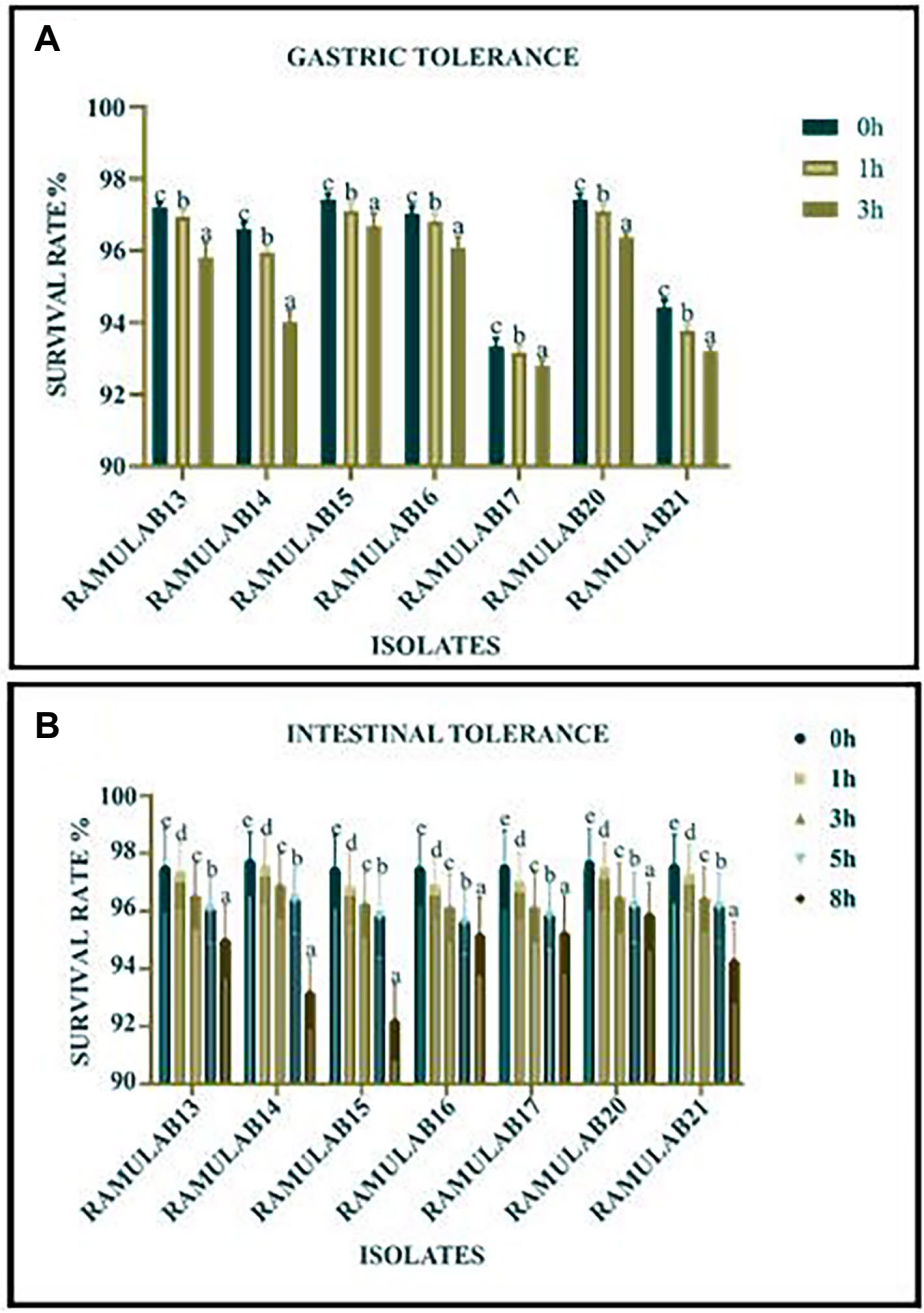
Survival rate (%) of gastric **(A)** and intestinal juice **(B)** tolerance of LAB strains after incubation for certain survival time intervals at 37°C. *Data are presented as mean ± standard deviation. Duncan’s multiple range tests revealed that the means of the survival rate with a 2 h time interval and superscripts **(a–e)** are substantially different from one another (*p* ≤ 0.05).

The growth of all seven isolates was optimal in both gastric and intestinal conditions. The isolates had a good survival rate up to a period of 8 h (Figures 2A,B). But with time, a slowing in growth was noticed. The plots depict the isolate’s tolerance levels for gastrointestinal conditions.

#### Resistance to phenol

The graph infers the tolerance of all the isolates towards 0.4% phenol (Table 2). The results showed a similar growth after incubation at different time intervals (0 and 24 h) with 0.4% phenol. The viable cell count varied between 6.74 and 7.52 Log CFU/mL. With 7.52 Log CFU/mL, the isolate RAMULAB15 demonstrated greater tolerance than other strains.

**Table 2:** Cell surface hydrophobicity (%) and phenol tolerance (Log CFU/mL) of the isolates.

Isolates	Cell-surface hydrophobicity (%)*	Phenol tolerance (Log CFU/mL)*	
		0 h	24 h
RAMULAB13	70.82 ± 7.5^b, c^	7.21 ± 0.10^b^	7.32 ± 0.22^b^
RAMULAB14	69.17 ± 9.8^b^	7.12 ± 0.21^a^	7.26 ± 0.31^a^
RAMULAB15	76.28 ± 8.4^d^	7.52 ± 0.60^d^	7.84 ± 0.41^e^
RAMULAB16	71.53 ± 2.5^c^	7.16 ± 0.12^a^	7.38 ± 0.24^c^
RAMULAB17	52.13 ± 3.4^a^	7.28 ± 0.25^c^	7.46 ± 0.32^d^
RAMULAB20	70.99 ± 5.8^b, c^	7.26 ± 0.42^c^	7.49 ± 0.12^d^
RAMULAB21	68.75 ± 10.4^b^	7.17 ± 0.42^a^	7.36 ± 0.14^c^

*Data are presented as mean ± standard deviation. Duncan’s multiple range tests revealed that the means of the survival rate with a 24 h time interval and superscripts (a-e) are substantially different from one another (*p* ≤ 0.05).

### Adherence assays

#### Cell surface hydrophobicity

Xylene was the polar solvent used for the determination of cell surface hydrophobicity. In the present study, RAMULAB15 and RAMULAB17 exhibited a maximum of 76.28% and a minimum of 52.13% hydrophobicity (Table 2).

#### Assay for auto and coaggregation

**Figure 3:**
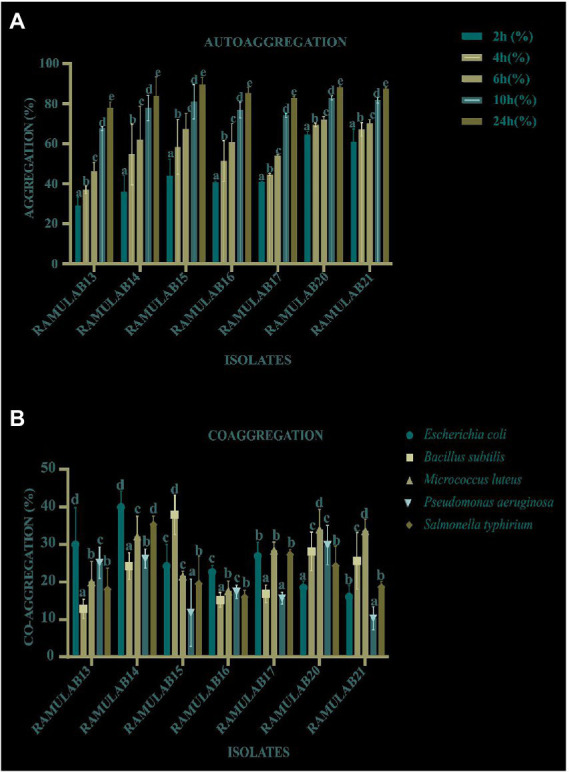
The autoaggregation (%) **(A)** and coaggregation **(B)** of LAB strains at different time intervals and 2 h incubation at room temperature. *Data are presented as mean ± standard deviation. Duncan’s multiple range tests revealed that the means of the survival rate with a 24 h time interval and superscripts **(a–e)** are substantially different from one another (*p* ≤ 0.05).

RAMULAB15 happened to show the highest autoaggregation percentage of 89.47% at 24 h. An exponential increase in the percentage of autoaggregation was observed in all the isolates over time between 2 and 24 h. All the isolates exhibited an autoaggregation activity >77% (Figure 3A). All seven isolates exhibited a high coaggregation percentage with *Micrococcus luteus* MTCC 1809. RAMULAB20 showed the highest coaggregation ability of 42.43% (Figure 3B). Probiotic autoaggregation and coaggregation are crucial for bacterial fortification and colonization and the present study clearly indicates the potential of the isolated LABs as probiotic alternatives.

#### Adhesion ability to chicken epithelial cells and HT 29 cell lines

**Figure 4:**
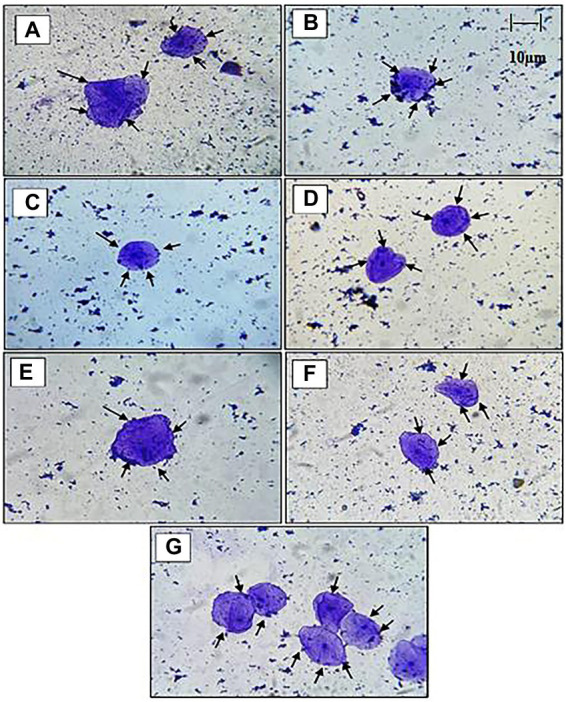
Under a light microscope, the adherence of LAB strains to chicken crop epithelial cells, The adhesion of isolates: **(A)** RAMULAB13, **(B)** RAMULAB14, **(C)** RAMULAB15, **(D)** RAMULAB16, **(E)** RAMULAB17, **(F)** RAMULAB20, **(G)** RAMULAB21 to chicken crop epithelial cells. LAB strains are seen adhering to the epithelial cells as indicated by the black arrow.

The adhesion capacity of the LAB isolated to chicken epithelial cells was determined to be between the minimum (18–25 cells) and (35–60 cells) maximum bacterial cells per epithelial cell (Figure 4). As indicated in Table 3, RAMULAB20 had the highest adhesion rate, whereas RAMULAB13 had the least. Isolates’ adherence to the HT-29 cells was greater than 79.82%. Comparatively, RAMULAB16 demonstrated the highest level of adhesion to the other isolates.

**Table 3:** Adhesion–measured by the percentage of isolates that adhere to HT-29 cells.

Isolates	Cell Adherence (%)*
RAMULAB13	79.82 ± 07.85 ^a^
RAMULAB14	81.17 ± 4.18 ^b^
RAMULAB15	83.28 ± 6.74 ^c^
RAMULAB16	88.53 ± 4.51 ^d^
RAMULAB17	85.13 ± 3.24 ^c^
RAMULAB20	80.99 ± 6.98 ^b^
RAMULAB21	86.75 ± 1.24 ^d^

*Data are presented as mean ± standard deviation. Duncan’s multiple range tests revealed that the means of the survival rate with a 24 h time interval and superscripts (a-e) are substantially different from one another (*p* ≤ 0.05).

### Safety assessment

#### Antibiotic sensitivity

To ascertain the profile of antibiotic resistance, the isolates were screened against 13 different antibiotics. The outcomes were compared to the reference standard chart. One of the basics is the assessment of probiotic qualities and antibiotic sensitivity. Erythromycin, chloramphenicol, rifampicin, gentamicin, ampicillin, tetracycline, streptomycin, clindamycin, and azithromycin were effective against the seven isolates. Conversely, vancomycin, methicillin, kanamycin, and cefixime were resistant (Table 4).

**Table 4:** Antibiotic susceptibility test of the isolates representing resistance and sensitivity based on CLSI, 2018.

Isolates	Antibiotics												
	E	C	RIF	GEN	AMP	TET	STR	CD	AZM	MET	K	CEF	VA
RAMULAB13	S	S	S	S	S	S	S	S	S	R	R	R	R
RAMULAB14	S	S	S	S	S	S	S	S	S	R	R	R	R
RAMULAB15	S	S	S	S	S	S	S	S	S	R	R	R	R
RAMULAB16	S	S	S	S	S	S	S	S	S	R	R	R	R
RAMULAB17	S	S	S	S	S	S	S	S	S	R	R	R	R
RAMULAB20	S	S	S	S	S	S	S	S	S	R	R	R	R
RAMULAB21	S	S	S	S	S	S	S	S	S	R	R	R	R

Erythromycin (E), Chloramphenicol (C), Rifampicin (RIF), Gentamicin (GEN), Ampicillin (AMP), Tetracycline (TET), Streptomycin (STR), Clindamycin (CD), Azithromycin (AZM), Methicillin (MET), Kanamycin (K), Cefixime (CEF), and Vancomycin (V).

#### Hemolytic and DNase assay

The six LAB isolates were confirmed safe and categorized under γ - hemolysis after 48 h of incubation at 37°C with no zone surrounding the colonies. This indicates that the isolates were safe to use, which constitutes another indicator of the probiotic formulation’s safety. Furthermore, DNase activity also fulfils as an indicator of the safety of the isolates. In the present study, the non-pathogenic nature of the isolates that showed no zone of inhibition was established.

### Antimicrobial activity

The antibacterial activity was evaluated against microbial pathogens. Significantly, all of the indicator bacteria were resistant to the isolate’s antimicrobial activity. The scale of the zone of inhibition is between 6 and 20 mm. All of the isolates showed an effective antibacterial potential against the opportunistic pathogens *P. aeruginosa* and *M. luteus*. RAMULAB15 had strong antimicrobial efficacy against all the pathogens tested except for *B. cereus, S.typhimurium, K. pneumonia*, and *K. aerogenes*. With the exception of a moderate inhibitory effect on the pathogens *S. aureus, P. aeruginosa*, and *M. luteus*, the isolate RAMULAB17 exhibited negligible activity against all the pathogens (Table 5).

**Table 5:** Antimicrobial activity of the LAB isolates against pathogens.

Pathogens	Isolates						
	RAMU LAB13	RAMU LAB14	RAMU LAB15	RAMU LAB16	RAMU LAB17	RAMU LAB20	RAMU LAB21
*M. luteus*	+++	+++	+++	+++	+++	+++	+++
*P. aeruginosa*	+++	++	+++	+++	++	+++	+++
*S. aureus*	++	++	++	++	++	++	++
*B. cereus*	+	−	+	+	−	+	+
*E. coli*	++	+	++	+	+	+	+
*B. subtilis*	++	+	++	+	−	++	+
*K. pneumoniae*	+	−	+	+	−	−	−
*S. typhimurium*	−	+	+	+	−	+	+
*K. aerogenes*	−	+	+	+	−	+	+
*P. florescens*	++	++	++	++	+	++	++

Symbols show zones of inhibition (mm): −, no inhibition; +, weak (< 7); ++, good (9–15); +++, strong (> 15).

### Molecular character and phylogenetic analysis

**Figure 5:**
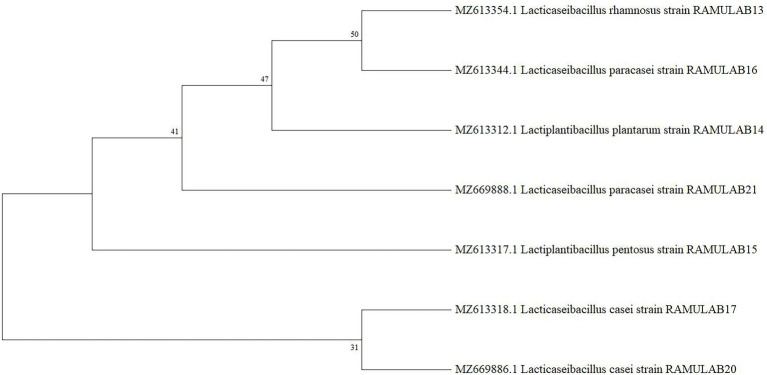
Analysis of the phylogenetic relationships among LAB isolates, based on 16S rRNA maximum likelihood bootstrap analysis.

The amplified 16S rRNA sequences of seven LAB isolates from fermented batters were subjected to evolutionary analyses using the MEGA X software. The homology search revealed that the sequences of strains RAMULAB13, RAMULAB14, RAMULAB15,RAMULAB16, RAMULAB17, RAMULAB20, and RAMULAB21 were > 95% similar to *Lacticaseibacillus rhamnosus, Lactiplantibacillus plantarum, Lactiplantibacillus pentosus, Lacticaseibacillus paracasei, Lacticaseibacillus casei, Lacticaseibacillus casei*, and *Lacticaseibacillus paracasei* respectively, thus validating the homology sequences of the isolates (Figure 5). Table 6 lists the NCBI GenBank accession number of all the isolates.

**Table 6:** Identified LAB isolates GenBank accession numbers.

Isolates	Sample	Bacteria	GenBank Accession no.
RAMULAB13	Jalebi batter	*Lacticaseibacillus rhamnosus*	MZ613354
RAMULAB14		*Lactiplantibacillus plantarum*	MZ613312
RAMULAB15		*Lactiplantibacillus pentosus*	MZ613317
RAMULAB16	Kallappam batter	*Lacticaseibacillus paracasei*	MZ613344
RAMULAB17		*Lacticaseibacillus casei*	MZ613318
RAMULAB20	Vada batter	*Lacticaseibacillus casei*	MZ669886
RAMULAB21		*Lacticaseibacillus paracasei*	MZ669888

### Antioxidant assay

**Figure 6:**
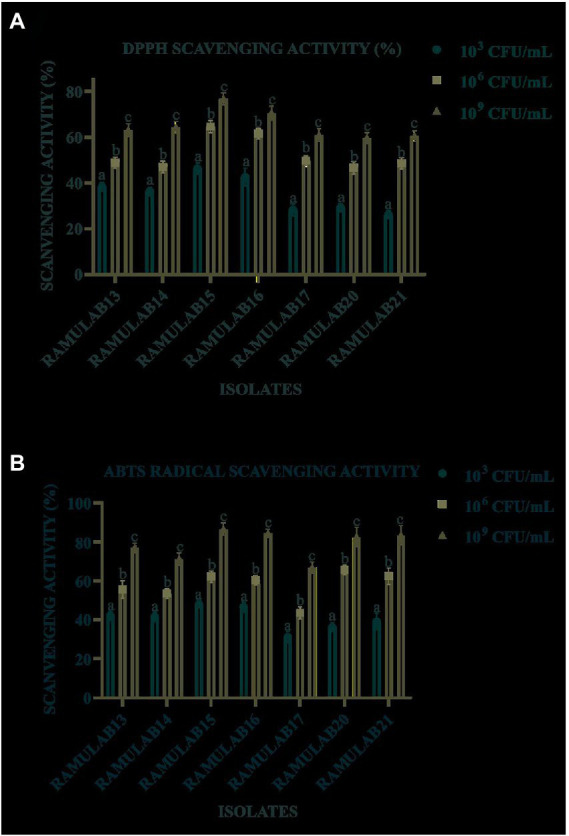
Isolates scavenging activity by the isolate on free DPPH **(A)** and ABTS **(B)** radicals. Results are presented as mean ± SD. Duncan’s multiple range test shows that the means of the same column that are denoted by different letters **(a–d)** are substantially different (*p* ≤ 0.05).

As the number of cells increased exponentially, all the isolates expressed a higher DPPH free radical scavenging activity in 10^9^ CFU/ml, with the results ranging from 26.12 to 76.63% (Figure 6A). RAMULAB15 exhibited the highest radical-scavenging activity (76.63%). At 10^9^ CFU/ml, the ABTS radical scavenging activity of the isolates ranged from 31.15 to 84.45%. RAMULAB17 and RAMULAB15 exhibited the lowest and highest inhibition activities, respectively (Figure 6B).

### Inhibitory assay for the carbohydrate hydrolyzing enzymes

**Figure 7:**
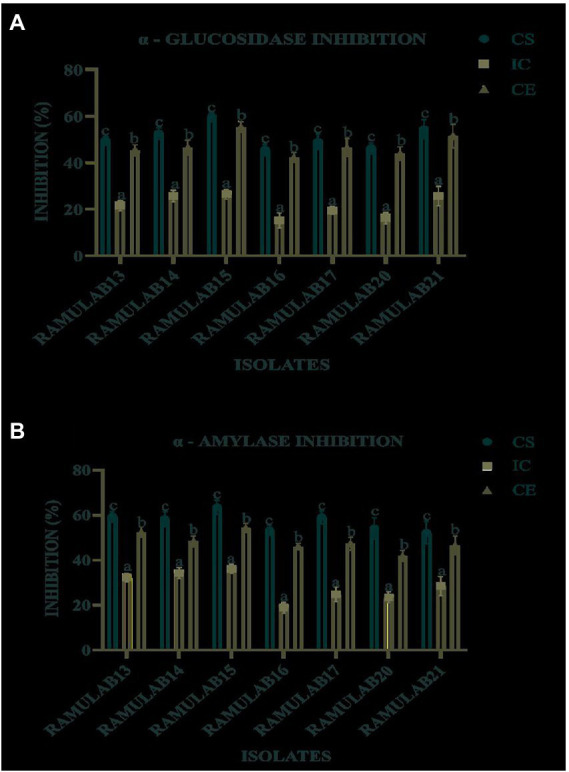
α-Glucosidase **(A)** and α-amylase **(B)** inhibitory activity of the isolates. Results are presented as mean ± SD. Duncan’s multiple range test shows that the means of the same column that are denoted by different letters **(a–d)** are substantially different (*p* ≤ 0.05).

The study used CS, CE, and IC obtained from the seven isolates to measure their inhibitory activity against α-glucosidase and α-amylase. For all isolates, the CS and CE exhibited a marked influence on both α-glucosidase and α-amylase. Inhibition of α-glucosidase by the isolates tested using CS, CE, and IC ranged from 15.08 to 59.55% (Figure 7A), whereas α-amylase inhibition ranged from 18.79 to 63.42% (Figure 7B). Strain RAMULAB15 exhibited the highest inhibition rate of 59.55 and 63.42% for both α-glucosidase and α-amylase, respectively. The intact cells from the isolates exhibited the least inhibition compared to the supernatant and pellets (Table 7; Figure 7).

**Table 7:** α-Glucosidase and α-Amylase inhibitory activity of the isolates.

Isolates	α-Glucosidase inhibition*	α-Amylase inhibition*				
	CS	IC	CE	CS	IC	CE
RAMULAB13	49.35 ± 1.97^b^	21.50 ± 2.23^b^	45.38 ± 2.34^b^	59.35 ± 2.34^c^	32.25 ± 1.97^d^	52.38 ± 2.23^d^
RAMULAB14	52.42 ± 2.47^c^	25.57 ± 2.45^c^	46.41 ± 3.24^c^	58.21 ± 3.24^c^	34.24 ± 2.47^e^	48.41 ± 2.45^c^
RAMULAB15	59.55 ± 1.84^d^	26.35 ± 2.06d	55.24 ± 2.24^e^	63.42 ± 3.24^e^	36.12 ± 1.84^e^	54.24 ± 2.06^e^
RAMULAB16	45.40 ± 2.45^a^	15.08 ± 3.20^a^	42.12 ± 2.14^a^	53.12 ± 2.14^b^	18.79 ± 2.45^a^	46.12 ± 1.42^b^
RAMULAB17	48.81 ± 3.24^b^	19.38 ± 1.84^b^	46.35 ± 3.87^c^	59.10 ± 2.87^d^	24.78 ± 3.24^b^	47.35 ± 2.84^c^
RAMULAB20	46.07 ± 2.42^a^	16.08 ± 2.45^a^	43.81 ± 2.95^a^	54.07 ± 4.95^b^	23.45 ± 2.42^b^	41.81 ± 2.45^a^
RAMULAB21	54.26 ± 4.24^c^	25.57 ± 4.21^c^	51.42 ± 4.98^d^	52.26 ± 4.98^a^	28.45 ± 4.24^c^	46.42 ± 4.21^b^

*Results are presented as mean ± SD. Duncan’s multiple range test shows that the means of the same column that are denoted by different letters (a–d) are substantially different (*p* ≤ 0.05).

## Discussion

A product or food’s sensory qualities can be affected by fermentation in addition to its significant potential to improve sustainability, nutrition, and safety leading to health benefits (Lorn et al., 2021). Although traditional spontaneous fermentation typically causes hindrance in certain acceptability features, choosing starters based on particular enzymatic activities can advance a clean label and natural oriented fermentation that results in food with particular sensory properties (Klongklaew et al., 2022). Traditional fermented batters and other types of food involving fermentation were discovered to be rich sources of probiotics based on prior research (Mullish et al., 2021; Grujović et al., 2022; Manovina et al., 2022). The probiotics isolated from these fermented sources have proven to exhibit antihyperglycemic properties (Ayyash et al., 2020; Lakra et al., 2020; Sarkar et al., 2020; Pereira et al., 2021) 0.9 percent of adults worldwide are affected with DM, a systemic chronic disease that can cause difficulties to the majority of the body’s organs (Cree-Green et al., 2012). Changes in the pancreatic beta-cell functions lead to insulin deficiency, which in turn results in the hindered cellular response to insulin production. One of the most notable effects of these events is an elevated blood glucose (hyperglycemia), which leads to a number of issues associated with diabetes. These events also induce several anomalies in physiological pathways (Poretsky, 2010). Ineffectiveness and unfavorable side effects are occasionally associated with the use of chemical treatments to regulate blood sugar. As a result, contemporary studies investigated earlier suggest other possible biotherapeutic therapies to be implemented involving probiotics (Akbari and Hendijani, 2016). Recent studies have discovered that diabetic patients had altered gut microbiota; Blood samples from diabetes individuals revealed the presence of circulating gram-positive gut bacteria (Sato et al., 2014). Through the release of lipopolysaccharides, the altered gut microbiota may cause metabolic endotoxemia, which in turn may promote insulin resistance and inflammation (Bekkering et al., 2014). A positive or negative correlation was found between the quantity of epithelial enteroendocrine L cells and the prevalence of 25 bacterial taxa in the intestine, which play a role in the development of inflammation (Everard and Cani, 2013). The impact of probiotic supplementation on diabetes and its associated ailments was examined by researchers in light of these findings. The therapeutic impact of probiotics on glycaemic control has been the subject of various investigations in humans. Recent clinical studies have produced encouraging outcomes, which emphasize the necessity of a thorough systematic review and meta-analysis of these results (Markowiak and Ślizewska, 2017; Kim et al., 2019; Dudek-Wicher et al., 2020; Montassier et al., 2021; Mullish et al., 2021; Quigley, 2022).

In the current study, the probiotic *Lactobacillus* spp. isolated from fermented batters (jalebi, medhu vada, and kallappam) are tested for their ability to inhibit the carbohydrate hydrolyzing enzymes α-glucosidase and α-amylase. Seven (07) isolates were chosen for further evaluation out of twenty-four (24) isolates that were retrieved based on their phenotypic traits. The following LAB strains were selected for further testing: 3 LAB strains [RAMULAB (13, 14, and 15)] from jalebi batter, 2 LAB strains [RAMULAB (16, 17)] from kallappam batter, and the rest 2 LAB strains [RAMULAB (20, 21)] from medhu vada batter were retrieved. At various temperatures as well as with regard to salt and acid bile tolerance, the viability of the seven LAB isolates was assessed considering them as important parameters in the probiotic evaluation. The viability rate at various temperatures, salt tolerance, and acid bile tolerance was >93% compared to earlier investigations (Al Kassaa et al., 2014; Reuben et al., 2020). Another key factor for sustaining numerous health benefits is the capacity of the isolates to survive under extreme pH circumstances comparable to that of the gastrointestinal tract. The gastrointestinal assay, which simulates the digestion of food in the stomach and intestine with pH levels of 2 and 8, for durations of 2–3 h and 3–8 h, respectively, was carried out to assess the probiotic potential (Gupta and Tiwari, 2015). For both of the simulated gastrointestinal tolerance conditions, all seven isolates exhibited a > 92% survival rate. A similar survival rate (%) was reported in accordance with the study examined by Kumari et al. (2022a,b). At pH 2, *P. pentosaceus SP2* and *L. paracasei SP5* both strains’ viability (%) was observed to be decreased (Mantzourani et al., 2019). In contrast to earlier studies, ours had a consistently high survival rate and a higher tolerance to bile acids, and gastrointestinal conditions. The phenolic conditions that can be created by bacterial deamination of amino acids generated from dietary proteins are able to support the survival of the gut microbiota. Phenol and other hazardous metabolites released during specific digestive processes can be produced by gut microbes. Any bacteria that can endure these conditions can therefore be considered to have probiotic potential (Billah et al., 2010; Padmavathi et al., 2018). After 24 h of incubation with 0.4% phenol, Jena et al. (2013) reported a progressive increase in the cell viability from 5.69 to 7.05 Log CFU/mL was observed previously. Similarly in our study, the cell viability increased from 7.12 to 7.84 log CFU/mL with 0.4% phenolic concentration after 24 h incubation, demonstrating cell viability of LAB with resistance to phenol in the GI tract. With a 7.84 Log CFU/mL viable count, strain RAMULAB15 demonstrated the maximum tolerance to 0.4% phenol in our study. RYPR1 and RYPC7 expressed the maximum tolerance to phenol with 7.73 and 7.39 Log CFU/mL (Yadav et al., 2016). In our study, it was discovered that the isolates were highly effective at tolerating phenol, and could survive along the transit of the gastrointestinal tract. In comparison with other investigations mentioned above, all seven isolates from our study demonstrated better tolerance to phenolic conditions.

Colonization due to hydrophobicity, autoaggregation, and coaggregation play a vital role in the probiotic evaluation of LAB. In contrast to coaggregation, where they adhere intercellularly to various strains of bacteria, autoaggregation and hydrophobicity allow the microorganisms to adhere to the intestinal mucosa where they bind to colonies of the same group of microorganisms (del Re et al., 2000; Li et al., 2015). Bacterial attachment to the human intestinal layer is a complex event involving a variety of elements, including the charges of both human and bacterial cells, their hydrophobicity, extracellular polysaccharides, and proteins (cell surface). To acquire proper, potent, or irreversible adhesion, bacterial cells must get through all of these obstacles (Abdulla et al., 2014). The current adherence investigation found that the hydrophobicity, autoaggregation, and coaggregation were above 52.13, 77.48, and 42.43%, respectively. This event contributes to maintaining the favorable environment of the gut. The study investigated the adhesion of LAB to chicken crop epithelial cells and HT29 cells as well, and the outcomes were encouraging >80.99% and a maximum of 35–60 cells/bacterial epithelial cell adhesion were reported by the investigated LAB strains to HT29 cell attachment and chicken crop epithelial cells. Thus, aggregation constitutes the defense mechanism against anti-infection for the host (Somashekaraiah et al., 2019; Li et al., 2020; Jeong et al., 2021). Prior studies have revealed the ability of *Lactobacillus* spp. to competitively adhere and minimize inflammatory effects while inducing a higher state of change in the host intestinal epithelial cells’ defense system (Dhanani and Bagchi, 2013). Thus from our study, it can be concluded that the adherence capability of the LAB isolates was most efficient than the results which are already available from other earlier investigations.

Additionally, the present study assessed the isolated strains’ safety and development. To assess the safety of the LAB, tests for - antibiotic sensitivity, DNase activity, hemolytic assay, and antimicrobial were performed. The antibiotic sensitivity profile was assessed using the CLSI 2018 Scale of Clinical and Laboratory Standard Institute. According to the findings, each of the seven isolates was resistant to methicillin, vancomycin, kanamycin, and cefixime. These LAB isolates are commendable for their positive effects on enhancing intestinal health, particularly when LABs are given concurrently with antibiotics, which can fend off illnesses brought on by other pathogens (Iorizzo et al., 2022). From our findings in the study, it can be deduced that the following LAB strains can be administered along with other known antibiotics in treating illness. The DNase test was also carried out to examine the pathogenicity of bacteria that generate the DNase enzyme, which hydrolyzes DNA. The absence of DNase in the tested isolates was therefore confirmed, indicating their potential safety for use in fermentation. The isolated LAB strains were also found to be harmless after a hemolytic experiment indicated no hemolysis (Saroj et al., 2016). On the other hand, *L. rhamnosus* has been found to compromise the integrity of cellular membranes and cause ATP efflux, which results in pore development and reduces the growth of *M. luteus* (Li et al., 2020). By demonstrating membrane permeabilization activity, the bacteriocin produced from *L. plantarum ZJ316* had a promising antibacterial action against *M. luteus* (Jiang et al., 2018). In the current investigation, all seven isolates demonstrated a strong antibacterial activity when tested against *M. leuteus* MTCC 1809 and effective activity against the other pathogenic organisms. The seven isolates from our study can be used as antibacterial agents against *M. luteus*. All of the seven isolates were sequenced at the 16S rRNA region in an effort to characterize them molecularly. The isolates were identified as LAB based on the sequence data, and phylogenetic analysis using maximum likelihood bootstrap revealed that the isolated strains were RAMULAB13 *L. rhamnosus*, RAMULAB14 *L. plantarum*, RAMULAB15 *L. pentosus*, RAMULAB16 *L. paracasei*, RAMULAB17 *L. casei*, RAMULAB20 *L. casei*, and RAMULAB21 *L. paracasei*. A similar method was used in the identification of LAB strains *L. plantarum subsp. plantarum, L. pentosus, and L. plantarum subsp. argentoratensis.*, by Agaliya and Jeevaratnam (2013), Endres et al. (2021). The bacterial cell surface components are associated with intact cells’ capacity to scavenge free radicals. In line with several other earlier investigations, the isolates in our investigation demonstrated a higher scavenging activity. Free radical production has been linked to the pathogenesis and progression of diabetes (Alkalbani et al., 2019; Kim et al., 2021). Inflicting oxidative harm on biomolecules, hydroxyl and related radicals are the most hazardous reactive oxygen species. Antioxidants are converted into molecules that are irreversibly stable by DPPH and ABTS using their electrons or hydrogen atoms. Our findings are consistent with other investigations that showed intact cells from a few LAB isolates, including *P. pentosaceus R1* and *L. brevis R4*, had much stronger ABTS radical scavenging ability than cell-free extract and supernatant. Exopolysaccharides, Mn^2+^, bioactive compounds, antioxidant enzymes, NADH, NADPH, and other antioxidant molecules are present in LAB strains (Jiang et al., 2018).

It is possible to foretell the inhibition of glucose production and the progressive decrease in postprandial hyperglycemic blood glucose absorption in the small intestine by monitoring the activities of the enzymes α-glucosidase and α-amylase (Ramu et al., 2014; Martiz et al., 2022). The main objective of the study is to assess the ability of the probiotic isolates to inhibit the carbohydrate metabolism enzymes α-glucosidase and α-amylase. According to Muganga et al. (2015) strains CCFM147 and CCFM240 showed strong inhibition of 27.9 and 32.9% for α-glucosidase and α-amylase, respectively. Also, the strains CCFM4 and CCFM240 notably showed the highest levels of inhibition for CE, at 11.8 and 3.3%, respectively. Water-soluble extracts of isolates KX881777, KX881772, and KX881779 reported by Alkalbani et al. (2019) exhibited >34% α-amylase inhibition. The strains indicated 3.9% α-glucosidase inhibitory activity, which is 2.5 times more than the results for acarbose (Kwun et al., 2020). In comparison with our study, the strains isolated from dosa batter exhibited an α-glucosidase inhibitory activity ranging from 7 to 65% (Kumari et al., 2022b). Strain TKSP 24 isolated from Korean fermented soybean sauce-Doenjang exhibited an α-glucosidase inhibitory activity ranging from 58 to 62% (Shukla et al., 2016). *L. brevis* KU15006’s CFS and CE had the highest levels of α-glucosidase inhibitory activity when compared to commercially available LAB, at 24.11 and 10.56% percent, respectively (Son et al., 2017). α-glucosidase inhibitory activity for 12 LAB species was examined by Chen et al. (2014), who found that 12 strains showed between 3.42–29.57% inhibition, although MKHA15’s activity was substantially stronger at 99.25%. It is noteworthy that HAGB was significantly greater than LPGB produced by the same *L. plantarum* spp. (Jang and Kim, 2021). According to the study findings by Dilna et al. (2015) *Lactobacillus plantarum RJF4* displayed cholesterol lowering property (42.24%) and a-amylase inhibition (40%), However, in our study, the CS of the isolates exhibited 59.55 and 63.42% inhibition potential for α-glucosidase and α-amylase enzymes, respectively. In comparison with earlier research mentioned above, our findings indicate that the isolate from our LAB strain RAMULAB15 exhibited higher inhibition potential towards both α-glucosidase and α-amylase. Therefore, it can be concluded that the inhibition by CS of the isolates could be used as an effective inhibitor of both α-glucosidase and α-amylase enzymes. The inhibition of intestinal α-glucosidase and α-amylase by the CS of the isolates can aid in the treatment of postprandial hyperglycemia and can reduce blood glucose levels. The inhibitory activity of α-glucosidase and α-amylase by the isolates in this regard can delay the onset of diabetes complications.

## Conclusion

Diabetes being a global epidemic, a constant increase in the proportion of cases attributed to western eating habits is turning into a serious problem. The goal of the current study is to assess the potential probiotic *Lactobacillus spp.* which was isolated from traditional fermented batters (jalebi, medhu vada, and kallappam), for use in the treatment of diabetes. Based on analysis of the phenotypic, biochemical, and molecular characterization, all seven isolates were identified as LAB. The strains demonstrated noteworthy results in terms of acid-bile, gastrointestinal tolerance, auto-and coaggregation abilities, antibiotic, hydrophobicity, and antibacterial features which are essential parameters to qualify them as probiotics. The seven LAB displayed significant inhibitory α-glucosidase and α-amylase activity. However, the isolates’ CS and CE showed more inhibitory action than the isolates’ IC when investigated. As a result, the probiotic isolates from fermented batters are a good source of possible anti-diabetic properties. Following purification, the isolates can be utilized as supplements. Additional *in vivo* research is required for further assessment.

## Data availability statement

The datasets presented in this study can be found in online repositories. The names of the repository/repositories and accession number(s) can be found in the article/supplementary material.

## Author contributions

RR planned and conceptualized the manuscript. VC and SH were involved in data analysis and method development. TA, SK, CC, and RA were involved in supervision, editing, and preparation of the manuscript draft. All authors contributed to the article and approved the submitted version.

## Conflict of interest

SK was employed by Kerry Food Center, Inc. CC was employed by Midwest Veterinary Services, Inc.

The remaining authors declare that the research was conducted in the absence of any commercial or financial relationships that could be construed as a potential conflict of interest.

## Publisher’s note

All claims expressed in this article are solely those of the authors and do not necessarily represent those of their affiliated organizations, or those of the publisher, the editors and the reviewers. Any product that may be evaluated in this article, or claim that may be made by its manufacturer, is not guaranteed or endorsed by the publisher.
